# Using Machine Learning to Predict Unplanned Hospital Utilization and Chemotherapy Management From Patient-Reported Outcome Measures

**DOI:** 10.1200/CCI.23.00264

**Published:** 2024-04-26

**Authors:** Zuzanna Wójcik, Vania Dimitrova, Lorraine Warrington, Galina Velikova, Kate Absolom

**Affiliations:** ^1^ UKRI Centre for Doctoral Training in Artificial Intelligence for Medical Diagnosis and Care, University of Leeds, Leeds, United Kingdom; ^2^ School of Computing, University of Leeds, Leeds, United Kingdom; ^3^ Leeds Institute of Medical Research, University of Leeds, St James's University Hospital, Leeds, United Kingdom; ^4^ Leeds Cancer Centre, Leeds Teaching Hospitals NHS Trust, Leeds, United Kingdom; ^5^ Leeds Institute of Health Sciences, University of Leeds, Leeds, United Kingdom

## Abstract

**PURPOSE:**

Adverse effects of chemotherapy often require hospital admissions or treatment management. Identifying factors contributing to unplanned hospital utilization may improve health care quality and patients' well-being. This study aimed to assess if patient-reported outcome measures (PROMs) improve performance of machine learning (ML) models predicting hospital admissions, triage events (contacting helpline or attending hospital), and changes to chemotherapy.

**MATERIALS AND METHODS:**

Clinical trial data were used and contained responses to three PROMs (European Organisation for Research and Treatment of Cancer Core Quality of Life Questionnaire [QLQ-C30], EuroQol Five-Dimensional Visual Analogue Scale [EQ-5D], and Functional Assessment of Cancer Therapy-General [FACT-G]) and clinical information on 508 participants undergoing chemotherapy. Six feature sets (with following variables: [1] all available; [2] clinical; [3] PROMs; [4] clinical and QLQ-C30; [5] clinical and EQ-5D; [6] clinical and FACT-G) were applied in six ML models (logistic regression [LR], decision tree, adaptive boosting, random forest [RF], support vector machines [SVMs], and neural network) to predict admissions, triage events, and chemotherapy changes.

**RESULTS:**

The comprehensive analysis of predictive performances of the six ML models for each feature set in three different methods for handling class imbalance indicated that PROMs improved predictions of all outcomes. RF and SVMs had the highest performance for predicting admissions and changes to chemotherapy in balanced data sets, and LR in imbalanced data set. Balancing data led to the best performance compared with imbalanced data set or data set with balanced train set only.

**CONCLUSION:**

These results endorsed the view that ML can be applied on PROM data to predict hospital utilization and chemotherapy management. If further explored, this study may contribute to health care planning and treatment personalization. Rigorous comparison of model performance affected by different imbalanced data handling methods shows best practice in ML research.

## Teaser

Using patient-reported data to improve ML models predicting hospital utilization during chemotherapy.

## INTRODUCTION

Cancer treatment side effects frequently negatively affect patients' health and often cause emergency hospitalization.^1,2^ Unplanned health care utilization can be detrimental for patients' physical and emotional well-being and can reduce health care quality through burdening health care systems.^3^ Early identification of factors contributing to acute hospital presentations can support planning for emergency admissions, increase the quality of care, and reduce health care costs.^2,4^ Predicting the risk of chemotherapy-related hospital utilization could also help personalizing cancer treatment decisions.^5,6^

**CONTEXT**

**Key Objective**
To assess if patient-reported outcome measures (PROMs) improve performance of machine learning (ML) models predicting hospital admissions, triage events, and changes to chemotherapy.
**Knowledge Generated**
PROMs improve ML models predicting unplanned hospital utilization and chemotherapy management. ML methods provide good performance predicting changes to chemotherapy.
**Relevance *(J.L. Warner)***
This study is one of the first published by this journal to show an improvement in ML model performance from including patient-reported outcomes. The authors plan to involve patients and clinicians to assess their attitudes to ML-based prediction in their future work.*
*Relevance section written by *JCO Clinical Cancer Informatics* Editor-in-Chief Jeremy L. Warner, MD, MS, FAMIA, FASCO.

Machine learning (ML) adoption in medicine can aid clinical decisions, improving health care quality.^7^ ML methods have been applied to predict health outcomes, including postsurgery complications,^8^ stroke rehabilitation success,^9^ epilepsy,^10^ or mortality.^11^ ML models can also be successful in predicting hospital utilization. For instance, binary classifiers were used to robustly predict hospital admissions on the basis of emergency department triage information and patients' medical history.^12^ Furthermore, ML algorithms were applied to electronic health records (EHR) to predict chemotherapy-related hospital admissions.^5^

However, these models did not include any information gathered from patients about their own health and well-being. Therefore, current AI models process the clinical information well, without consideration of patients' perspective on their health.

Patient-reported outcome measures (PROMs) are questionnaires that measure patients' perception on their own health status,^13^ including disease-related symptoms, side effects of treatments, quality of life, and impact on functioning. PROMs are increasingly incorporated in routine clinical care and can be used as predictors in ML methods foreseeing health outcomes,^14,15^ for example, identifying patients at risk of experiencing undesirable clinical outcomes.^16^ ML algorithms trained on patient-reported and clinical data accurately predicted financial toxicity in patients with early breast cancer.^17^ Furthermore, PROMs enhanced ML performance predicting 5-year cancer survival, when added to clinical and sociodemographic variables.^18^ Nevertheless, the benefits of inclusion of PROMs as predictors are inconsistent, as some studies did not find PROMs to have as meaningful impact on model performance as objective measures.^19,20^

The variability in effectiveness of PROMs in predicting patient outcomes may be caused by inconsistent performance metrics and conclusions drawn from data affected by inappropriate preprocessing methods, such as balancing data sets before creating training and testing sets, which often introduces bias.^21^ The lack of methodologic agreement and guidance in the literature indicates the need for comparison of frequently used methods. The predictive value of PROMs is also not explored in detail because of the variety of PROMs currently used.^15^ Therefore, this paper aims to address five research questions:

1. Do PROMs add predictive value to ML models?
2. Which PROMs are the most useful in predictions?
3. Which ML models have the best performance?
4. Did preprocessing method for handling class imbalance affect model performance?
5. Which features were the most important for prediction?

## MATERIALS AND METHODS

### Data Set

Data from 508 patients initiating systemic treatment for colorectal, breast, or gynecologic cancers at Leeds Cancer Centre (United Kingdom), collected in an eRAPID clinical trial between January 22, 2015, and June 11, 2018,^22^ were used in this study. The data set contained 35 variables. Eight variables were clinical or demographic, collected from EHR. They included age at study entry, sex (male/female), number of days on study from the start of chemotherapy, study arm, disease site (breast/gynecologic/colorectal), previous chemotherapy (yes/no), information if the disease was metastatic or nonmetastatic, and the number of comorbidities (from the list: cardiovascular, respiratory, gastrointestinal, stomach/intestine, endocrine, renal, neurologic, rheumatologic, previous malignancy, and substance abuse). Twenty-four variables were from PROMs completed by participants on paper at the time of study entry. Fifteen of these PROMs were from European Organisation for Research and Treatment of Cancer Core Quality of Life Questionnaire (QLQ-C30)^23^ with 30 items, containing information about participants' physical symptoms, perception on their physical function, emotional and social function, and overall health and quality of life. Another five PROM variables were from Five-Dimensional Visual Analogue Scale (EQ-5D),^24^ including self-reported data on mobility, self-care, usual activities, pain/discomfort, and anxiety/depression. Four remaining PROM variables were aggregated scores of physical, social, emotional, and functional well-being from Functional Assessment of Cancer Therapy-General (FACT-G) 28 items.^25^ Three target variables were the number of hospital admissions, triage events (patients contacting emergency helpline or attending oncology admission unit), and changes to chemotherapy during the 18-week clinical trial. This information was extracted from EHR. The variables were selected because of their availability from the eRAPID clinical trial^22^ and the consultation with clinicians regarding their relevance.

### Variable Preparation

**FIG 1.**
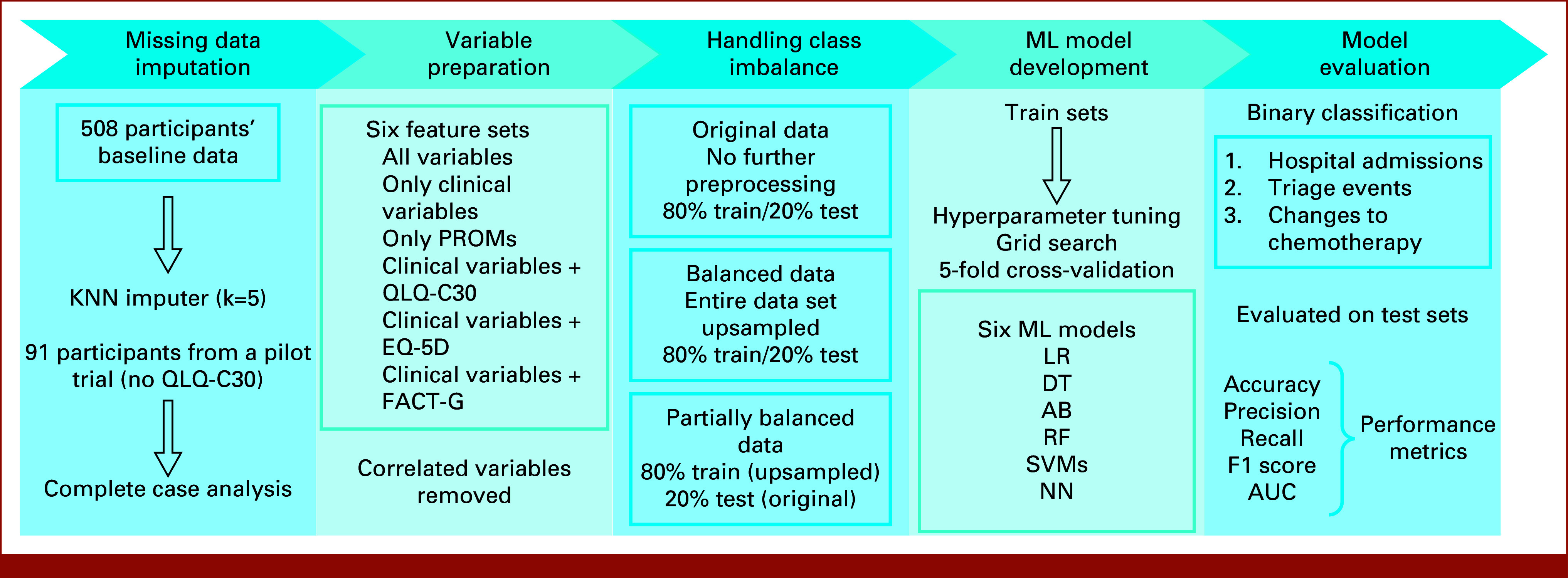
Flow diagram illustrating the methodology of the study. AB, adaptive boosting; DT, decision tree; EQ-5D, EuroQol Five-Dimensional Visual Analogue Scale; FACT-G, Functional Assessment of Cancer Therapy-General; KNN, k-nearest neighbors; LR, logistic regression; ML, machine learning; NN, neural network; PROMs, patient-reported outcome measures; QLQ-C30, European Organisation for Research and Treatment of Cancer Core Quality of Life Questionnaire; RF, random forest; SVMs, support vector machines.

The overview of the methods is presented in Figure 1. Target features were transformed to binary variables with class 0 (no event) or 1 (at least one event) to enable binary classification.^26,27^ To allow in-depth exploration of all PROM effects on the model performance in general, and when individual questionnaires are separately added to clinical data, six different feature sets were created with following variables:

1. Only clinical
2. All available
3. Only PROMs
4. Clinical + QLQ-C30
5. Clinical + EQ-5D
6. Clinical + FACT-G

Continuous variables were scaled to unit variance to improve computational performance of ML.^28^ To prevent algorithms from receiving repeated information,^29^ correlated variables were removed from each feature set (leaving one), so that no Pearson coefficient higher than 0.6 was left.^30^ The list of variables in each feature set is presented in Appendix Tables A1 and A2, including differences between classes.

### Missing Data Imputation

All patients completed QLQ-C30, EQ-5D, and FACT-G at the clinical trial baseline. However, for 91 participants whose data were taken from the pilot study of the trial, only two subscales of QLQ-C30 were included, so patients from this phase did not have full QLQ-C30 data. The records from these participants were removed from affected feature sets (all variables, only PROMs, and clinical + QLQ-C30 variables). Using complete case analysis (CC) is justified under the missing completely at random assumption. Pilot trial ensures random selection of participants, so CC method is unlikely to bias results.^31^ Any further cases of missing values were infrequent and likely resulted from participants omitting questions, which is a common issue in PROM data.^32^ They were imputed using K-nearest neighbors algorithm (*k* = 5), being a common imputation method in relevant studies.^18,26,33,34^

### Handling Class Imbalance

To mitigate potential bias of class imbalance,^21^ synthetic participants in minority class can be created to match the number of participants in the majority class (oversampling). In previous studies, it was performed on the entire data set^18,35^ or training set only.^36,37^ ML can also be trained on original data and evaluated using multiple performance metrics.^33^ Since there is no consistency in data preprocessing methods, the model performances in these scenarios were compared to discover bias in the results. Therefore, three data sets were created from each of the six feature sets for all target variables.

1. Original (no preprocessing method, 80% training set, 20% testing set)
2. Balanced (random sampling with replacement [oversampling] before train/test split with 8:2 ratio)
3. Partially balanced (train/test split with 8:2 ratio, stratification ensuring the same proportion of classes in both sets,^26^ oversampling performed on the training set, leaving testing set imbalanced)

### ML Model Development

Six ML models, namely, logistic regression (LR), decision tree (DT), adaptive boosting (AB), random forest (RF), support vector machines (SVMs), and neural network (NN), were selected on the basis of their inclusions in previous research.^18,37^ Hyperparameter tuning was performed on training sets through grid search with five-fold cross-validation.^37^ The models were applied using Python *sklearn* library.

### Model Evaluation

Accuracy, precision, recall (also known as sensitivity), F1 score, and AUC were used to evaluate model performance. AUC, a commonly used metric in ML studies, was considered a main metric for model evaluation to enable between-studies comparisons. Model calibrations were evaluated with calibration plots of RF in balanced data sets and LR in remaining data sets because of the best overall performance of these models in these scenarios. Feature importance analyses were also performed on these models. LR features were analyzed through the absolute values of regression coefficients. This method is only meaningful for standardized data with no multicollinearity,^38^ which was accounted for by standardization of features and removing correlated variables. RF features were explored through “*feature importances*” python command in *sklearn.RandomForestClassifier*. Analysis of variance with Tukey's honest significant difference tests were performed to compare model performances (Appendix Table A3).

### Clinical Feedback

Involving health professionals in early stages of exploratory research could support the clinical adoption of ML models.^39^ Therefore, the methodology design was finalized after feedback from the Patient Centred Outcomes Research Group in the University of Leeds, Faculty of Medicine and Health, Leeds Institute of Medical Research. This group includes oncologists, nurses, and psychologists.

## RESULTS

Performance metrics and hyperparameters for all models applied to all feature sets for all preprocessing methods are presented in Appendix Table A4.

### Hospital Admissions

#### Overall Predictive Value of PROMs

For all models in original and balanced data sets, clinical variables had worse AUC than feature sets including PROMs. In the partially balanced data set, F1 score was higher for clinical variables in SVM (0.188) and NN (0.493) than for other feature sets. Nevertheless, these values were not the highest overall. For SVM, recall was also the highest value for the clinical variables (0.176). No AUC was the highest for clinical variables.

#### Predictive Value of Individual PROM Questionnaires

In the original data set, clinical + QLQ-C30 variables achieved the best AUC in all models except NN (AUC was highest for PROMs only). In the balanced data set, clinical + QLQ-C30 variables obtained the highest AUC in all models, apart from LR and SVM (AUCs were highest for all variables). In the partially balanced data set, the highest AUC was obtained by clinical + QLQ-C30 variables in LR, DT, RF (the same value as PROMs only), and NN. In AB, the highest AUC was achieved by all variables, and in SVM by clinical + FACT-G variables. Therefore, QLQ-C30 variables aided ML performance the most.

#### Model Performance

**FIG 2.**
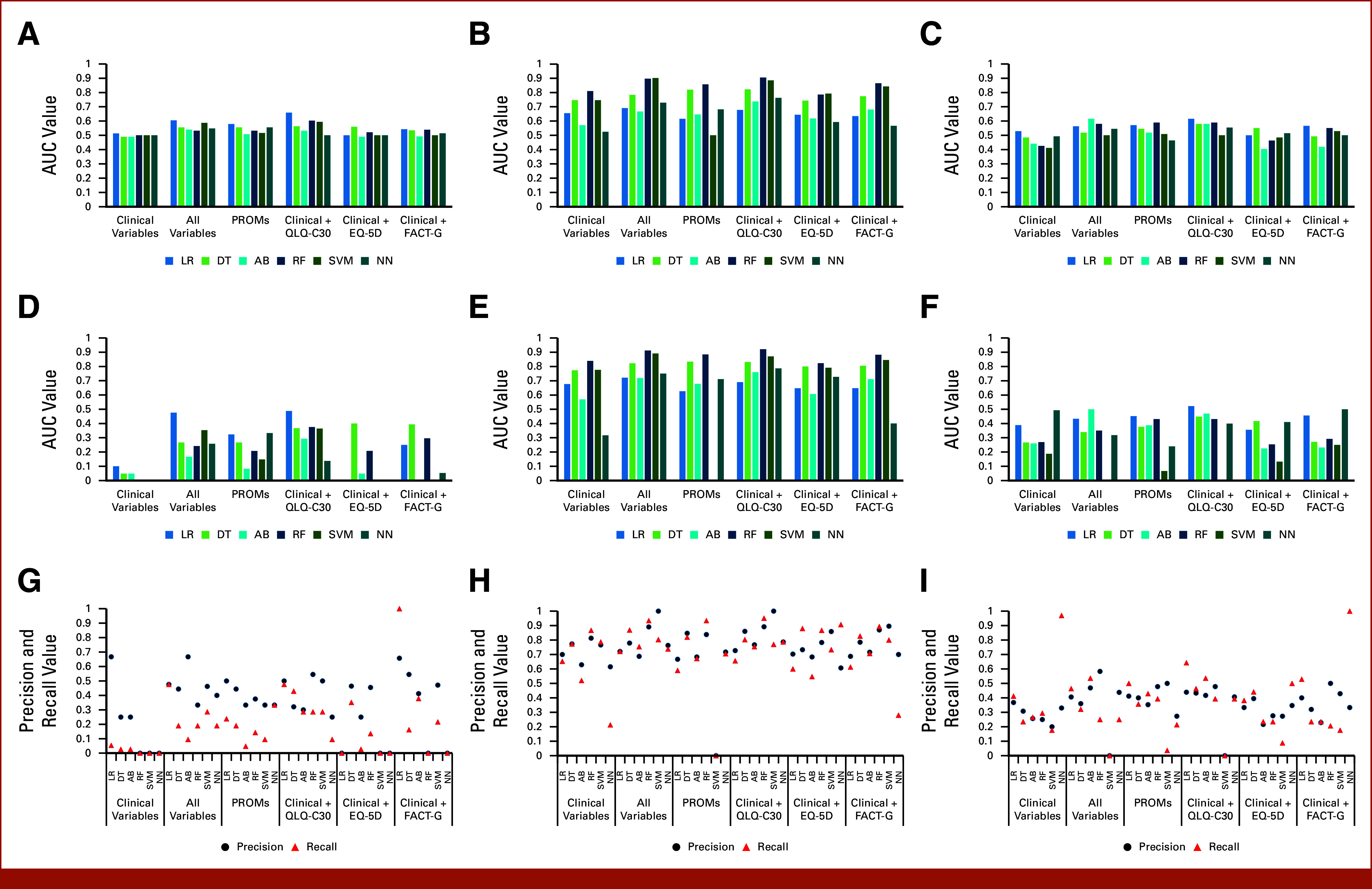
Predicting admissions. AUC values for (A) original, (B) balanced, and (C) partially balanced data sets obtained from all models in all feature sets; F1 scores for (D) original, (E) balanced, and (F) partially balanced data sets obtained from all models in all feature sets; precision and recall distributions for (G) original, (H) balanced, and (I) partially balanced data sets obtained from all models in all feature set. AB, adaptive boosting; DT, decision tree; EQ-5D, EuroQol Five-Dimensional Visual Analogue Scale; FACT-G, Functional Assessment of Cancer Therapy-General; LR, logistic regression; NN, neural network; PROMs, patient-reported outcome measures; QLQ-C30, European Organisation for Research and Treatment of Cancer Core Quality of Life Questionnaire; RF, random forest; SVMs, support vector machines.

**FIG 3.**
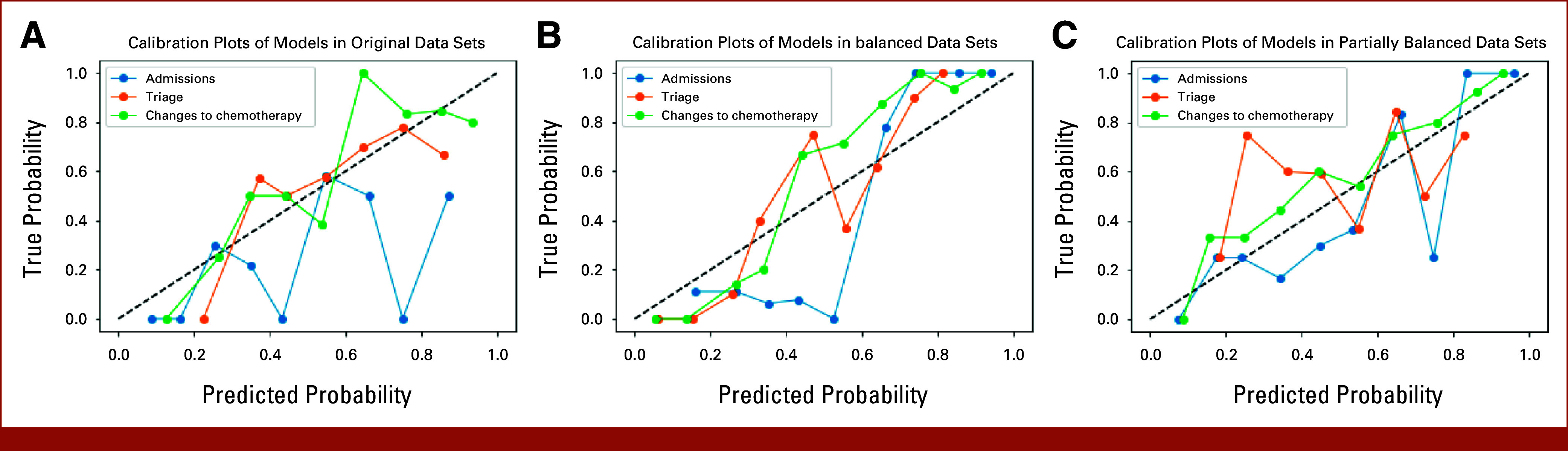
Calibration plots of LR models in original dataset (A), calibration of RF models in balanced datasets (B), and calibration of LR models in partially balanced datasets (C). LR, logistic regression, RF, random forest.

In the original data set, LR performed best in all feature sets, except for clinical + EQ-5D, where DT was superior (Fig 2A). The highest AUC (0.659) was obtained by LR with clinical + QLQ-C30 variables. In the balanced data set, RF was the best performing algorithm (highest AUC = 0.905) for all feature sets, apart from all variables and clinical + EQ-5D variables, where SVM performed slightly better. In the partially balanced data set, the best AUC (0.616) was achieved by LR using clinical + QLQ-C30 variables and AB using all variables. Balancing the entire data set improved model performance on the basis of all evaluation metrics (Figs 2B and 2E). Using partially balanced data resulted in similar AUCs and precision to original data, but improved F1 score and recall. Model calibration for predicting admissions is poor in original data and improves slightly in balanced and partially balanced scenarios (Fig 3). LR prioritized clinical variables, while RF focused on PROMs and age at study entry (Table 1).

**TABLE 1.** Feature Importance for LR and RF Models Predicting All Three Target Variables

Target Variable	Type of Variable	Input Variable	Original Data				Balanced Data				Balanced Train/Real Test			
			LR		RF		LR		RF		LR		RF	
			Coefficient	Rank	Value	Rank	Coefficient	Rank	Value	Rank	Coefficient	Rank	Value	Rank
Admissions	Clinical/demographic	AgeStudyEntry	0.005	19	0.052	5	–0.041	13	0.093	3	0.019	12	0.108	1
		StudyArm	0.060	11	0.006	23	0.100	12	0.013	22	0.085	9	0.021	20
		DiseaseSite	–0.348	4	0.019	17	–0.412	2	0.033	13	–0.404	3	0.032	15
		PreviousChemo	–0.402	2	0.003	24	–0.290	4	0.012	24	–0.241	4	0.012	23
		PrimaryorMet	0.866	1	0.037	13	1.047	1	0.044	9	0.732	1	0.033	12
		Comorbidities	0.366	3	0.034	14	0.335	3	0.039	10	0.484	2	0.055	6
		DaysonStudy	0.007	17	0.037	12	0.004	19	0.019	20	0.006	18	0.013	22
	QLQ-C30	C30_Appetite_0	0.008	16	0.106	3	0.011	15	0.050	5	0.008	16	0.036	10
		C30_Dyspnoea_0	0.013	14	0.128	2	0.013	14	0.048	7	0.017	14	0.042	8
		C30_NauseaVom_0	0.013	15	0.052	6	–0.003	24	0.017	21	0.006	19	0.017	21
		C30_Const_0	–0.003	20	0.009	21	–0.005	17	0.026	17	–0.002	23	0.033	14
		C30_Diarr_0	–0.006	18	0.013	20	–0.003	20	0.023	19	–0.004	21	0.024	19
		C30_Financ_0	–0.001	22	0.040	10	–0.007	16	0.030	15	–0.007	17	0.032	16
		C30_Cognitive_0	–0.001	23	0.030	15	–0.003	22	0.045	8	0.003	22	0.043	7
		C30_Sleep_0	–0.003	21	0.014	19	–0.003	21	0.035	12	–0.005	20	0.035	11
		C30_Social_0	0.000	24	0.039	11	0.005	18	0.048	6	–0.013	15	0.061	5
	EQ-5D	QoLEQ5DMob	–0.061	10	0.017	18	0.225	8	0.029	16	–0.002	24	0.030	17
		QoLEQ5DSelCar	0.197	6	0.055	4	0.112	11	0.013	23	–0.217	5	0.009	24
		QoLEQ5DUsuAct	0.165	8	0.043	9	–0.147	9	0.024	18	0.124	7	0.033	13
		QoLEQ5DPain	0.030	13	0.045	8	0.128	23	0.031	14	–0.132	6	0.029	18
		QoLEQ5DAnxDep	–0.039	12	0.009	22	0.239	7	0.037	11	–0.117	8	0.038	9
	FACT-G	PhysicalWB_Baseline	–0.188	7	0.132	1	–0.260	5	0.104	1	–0.017	13	0.094	2
		FunctionalWB_Baseline	0.163	9	0.051	7	0.126	10	0.102	2	–0.079	10	0.090	3
		SocialWB_Baseline	0.248	5	0.028	16	0.259	6	0.086	4	0.053	11	0.080	4
Triage	Clinical/demographic	AgeStudyEntry	–0.127	10	0.097	2	–0.186	8	0.119	1	–0.265	3	0.121	1
		StudyArm	–0.162	9	0.006	23	–0.202	7	0.014	22	0.243	4	0.026	17
		DiseaseSite	–0.166	8	0.013	22	–0.255	5	0.027	16	–0.196	7	0.030	15
		PreviousChemo	–0.721	1	0.019	19	–0.430	3	0.013	24	–0.643	1	0.022	20
		PrimaryorMet	0.487	2	0.004	24	0.644	1	0.018	20	0.624	2	0.017	23
		Comorbidities	0.338	5	0.044	10	0.397	4	0.036	11	0.108	9	0.036	10
		DaysonStudy	0.012	15	0.034	12	0.001	24	0.014	23	0.010	19	0.020	21
	QLQ-C30	C30_Appetite_0	0.006	18	0.077	3	0.002	23	0.032	12	0.000	24	0.031	14
		C30_Dyspnoea_0	0.003	23	0.028	16	0.004	21	0.026	17	0.015	16	0.019	22
		C30_NauseaVom_0	–0.005	20	0.031	13	0.009	16	0.019	19	0.002	22	0.022	19
		C30_Const_0	–0.006	17	0.016	20	–0.010	15	0.032	13	–0.017	15	0.034	13
		C30_Diarr_0	0.004	21	0.053	8	0.008	17	0.029	14	0.026	12	0.036	9
		C30_Financ_0	–0.004	22	0.019	18	–0.006	19	0.026	18	–0.010	20	0.029	16
		C30_Cognitive_0	–0.005	19	0.062	6	0.002	22	0.043	7	–0.009	21	0.044	7
		C30_Sleep_0	0.006	16	0.050	9	0.005	20	0.049	5	0.012	18	0.051	5
		C30_Social_0	–0.002	24	0.029	15	–0.008	18	0.049	6	–0.001	23	0.044	6
	EQ-5D	QoLEQ5DMob	0.024	13	0.013	21	–0.183	10	0.028	15	–0.115	8	0.026	18
		QoLEQ5DSelCar	–0.444	3	0.042	11	–0.207	6	0.014	21	–0.222	6	0.008	24
		QoLEQ5DUsuAct	0.283	6	0.056	7	0.088	14	0.037	9	0.021	13	0.034	12
		QoLEQ5DPain	0.053	12	0.030	14	0.440	2	0.040	8	0.019	14	0.035	11
		QoLEQ5DAnxDep	0.069	11	0.019	17	0.100	13	0.037	10	0.013	17	0.037	8
	FACT-G	PhysicalWB_Baseline	–0.258	7	0.122	1	–0.131	12	0.096	4	–0.234	5	0.091	3
		FunctionalWB_Baseline	0.364	4	0.074	4	0.186	9	0.103	2	0.074	10	0.096	2
		SocialWB_Baseline	0.013	14	0.063	5	0.171	11	0.099	3	0.036	11	0.090	4
Changes to chemotherapy	Clinical/demographic	AgeStudyEntry	–0.295	6	0.113	2	–0.138	6	0.112	2	–0.179	9	0.127	1
		StudyArm	–0.167	9	0.008	24	–0.137	7	0.016	22	–0.084	13	0.021	19
		DiseaseSite	1.007	1	0.128	1	–0.197	4	0.098	3	0.755	1	0.071	5
		PreviousChemo	0.034	12	0.012	23	–0.159	5	0.016	21	–0.226	6	0.015	22
		PrimaryorMet	–0.723	2	0.022	17	0.294	3	0.018	19	–0.299	3	0.018	20
		Comorbidities	0.572	3	0.064	6	0.316	2	0.031	12	0.396	2	0.038	10
		DaysonStudy	–0.002	21	0.024	16	0.000	24	0.014	23	0.003	23	0.009	24
	QLQ-C30	C30_Appetite_0	0.010	17	0.035	8	0.003	22	0.039	8	0.011	17	0.029	14
		C30_Dyspnoea_0	0.000	24	0.020	19	0.004	21	0.013	24	0.005	22	0.016	21
		C30_NauseaVom_0	0.009	18	0.019	21	0.008	16	0.021	18	0.037	15	0.021	19
		C30_Const_0	0.004	20	0.027	14	–0.010	15	0.026	14	–0.008	20	0.029	15
		C30_Diarr_0	–0.010	16	0.022	18	0.008	17	0.023	16	–0.011	18	0.028	16
		C30_Financ_0	–0.010	15	0.029	12	–0.006	19	0.032	11	–0.009	19	0.034	11
		C30_Cognitive_0	0.000	23	0.024	15	0.001	23	0.038	9	0.014	16	0.044	7
		C30_Sleep_0	0.001	22	0.044	7	0.005	20	0.040	7	0.006	21	0.041	9
		C30_Social_0	0.006	19	0.034	9	–0.007	18	0.046	6	–0.001	24	0.047	6
	EQ-5D	QoLEQ5DMob	0.241	7	0.033	10	–0.135	9	0.027	13	0.137	10	0.027	17
		QoLEQ5DSelCar	0.446	4	0.019	20	–0.129	10	0.017	20	0.282	4	0.013	23
		QoLEQ5DUsuAct	0.052	11	0.032	11	0.043	14	0.034	10	0.218	7	0.041	8
		QoLEQ5DPain	–0.211	8	0.029	13	0.334	1	0.024	15	–0.128	11	0.031	13
		QoLEQ5DAnxDep	–0.136	10	0.016	22	0.063	13	0.023	17	–0.056	14	0.031	12
	FACT-G	PhysicalWB_Baseline	–0.011	14	0.066	5	–0.125	11	0.064	5	0.274	5	0.079	4
		FunctionalWB_Baseline	–0.026	13	0.112	3	0.108	12	0.143	1	–0.091	12	0.109	2
		SocialWB_Baseline	–0.358	5	0.071	4	0.136	8	0.085	4	–0.215	8	0.082	3

Abbreviations: EQ-5D, EuroQol Five-Dimensional Visual Analogue Scale; FACT-G, Functional Assessment of Cancer Therapy-General; LR, logistic regression; QLQ-C30, European Organisation for Research and Treatment of Cancer Core Quality of Life Questionnaire; RF, random forest.

### Triage Events

#### Overall Predictive Value of PROMs

No AUC was highest for clinical variables in any of the models and data sets, suggesting that PROMs improved model performance. The only highest values obtained by only clinical variables were F1 score and recall in original data (NN) precision in balanced data (AB), but these were not the highest values considering all models.

#### Predictive Value of Individual PROM Questionnaires

In the original data set, feature sets achieving the highest AUCs were only PROM variables for LR, DT, and RF; all variables for SVM and NN; and clinical + QLQ-C30 variables for AB. In the balanced data set, all variables obtained the highest AUC for DT, AB, RF, and SVM. Clinical + QLQ-C30 variables had the highest performance for LG, and clinical + EQ-5D variables for NN. In the partially balanced data set, clinical + EQ-5D variables had the best performance the most frequently (for AB, RF, SVM, and NN), and clinical + FACT-G variables were selected twice (for LR and DT).

#### Model Performance

**FIG 4.**
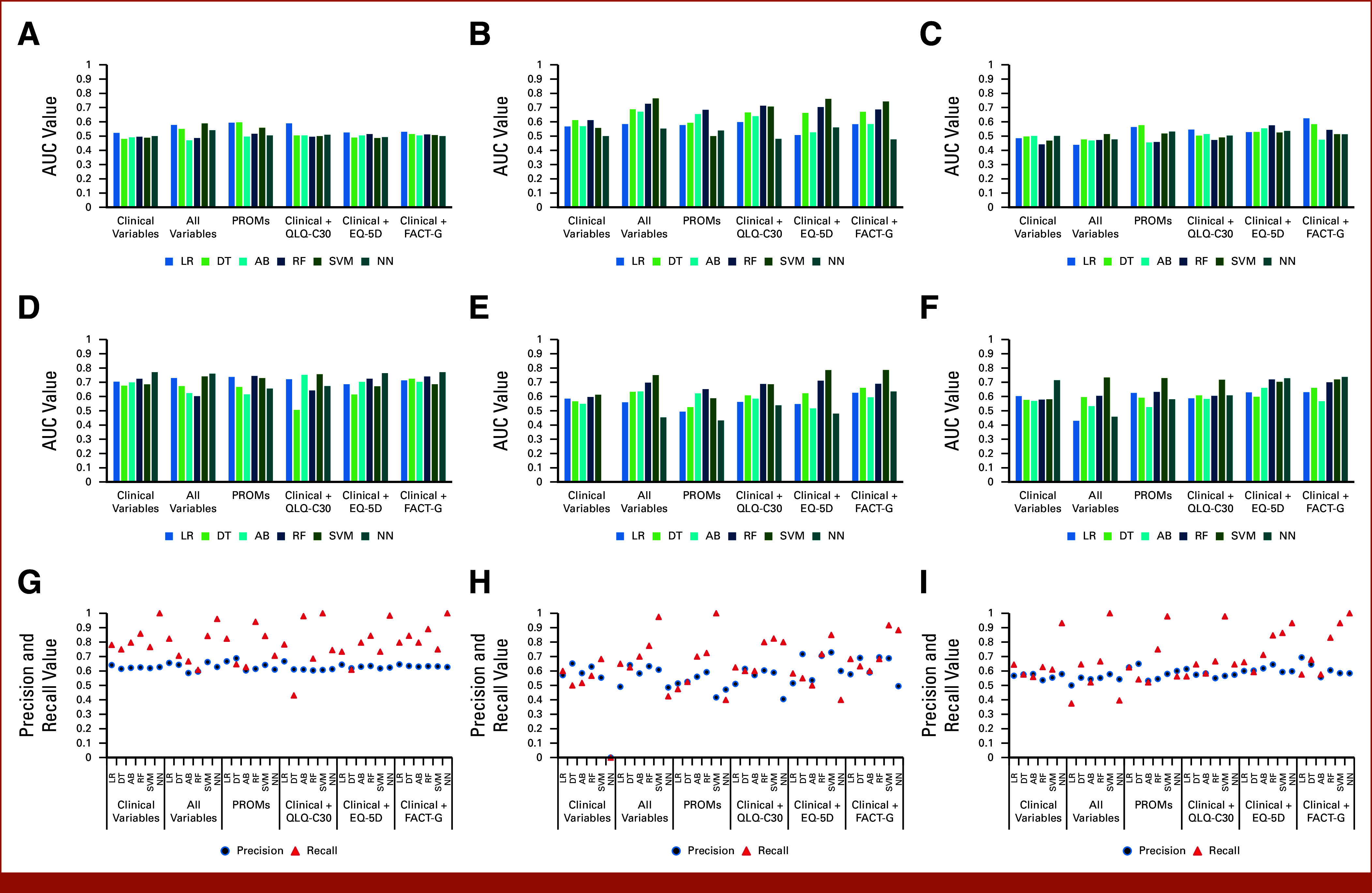
Predicting triage. AUC values for (A) original, (B) balanced, and (C) partially balanced data sets obtained from all models in all feature sets; F1 scores for (D) original, (E) balanced, and (F) partially balanced data sets obtained from all models in all feature sets; precision and recall distributions for (G) original, (H) balanced, and (I) partially balanced data sets obtained from all models in all feature sets. AB, adaptive boosting; DT, decision tree; EQ-5D, EuroQol Five-Dimensional Visual Analogue Scale; FACT-G, Functional Assessment of Cancer Therapy-General; LR, logistic regression; NN, neural network; PROMs, patient-reported outcome measures; QLQ-C30, European Organisation for Research and Treatment of Cancer Core Quality of Life Questionnaire; RF, random forest; SVMs, support vector machines.

Overall, models predicting triage performed significantly worse than models predicting changes to chemotherapy (*P* < .001) and admissions (*P* < .01). LR outperformed other models, achieving the highest AUC values across all feature sets, apart from PROMs only variables in original data, where DT achieved the best AUC. In the balanced data set, SVM and RF performed the best (highest AUC = 0.764 for SVM for all variables). In the partially balanced data set, the best AUC (0.624) was obtained by LR in the clinical + FACT-G feature set. There was no outstanding model in the original data set, but in balanced and partially balanced data sets, SVM, RF, and NN provided the best F1 scores (Figs 4E and 4F). A slight increase in the AUCs for the balanced data set is noticeable in Figure 4B. AUCs and F1 scores in the partially balanced data set were similar to the original data set. For some models in balanced data, the F1 scores were lower than in other data sets. Model calibration remained poor across different data types (Fig 3). LR's main features comprised clinical data with individual PROM variables, while RF primarily considered FACT-G variables (Table 1).

### Changes to Chemotherapy

#### Overall Predictive Value of PROMs

In the original data set, AUC was the highest for clinical variables only in DT (0.623) and RF (0.623). However, these values were not the highest overall. In the balanced data set, recall was the only measure highest for clinical variables in AB (0.754) and NN (1). In the partially balanced data set, clinical variables obtained the highest precision (0.872) and AUC (0.682) in LR and SVM, respectively. Overall, highest AUC had models including PROMs.

#### Predictive Value of Individual PROM Questionnaires

In the original data set, clinical + QLQ-C30 variables had the highest performance in LR and SVM; clinical + FACT-G variables for AB and NN; and clinical variables for DT and RF. In the balanced data set, clinical + QLQ-C30 variables obtained the highest AUC in DT and AB, clinical + EQ-5D variables for NN, and all variables for LR and RF. In SVM, all variables and clinical + QLQ-C30 variables achieved the same AUC (0.931). In the partially balanced data set, all variables obtained the highest AUC for DT, AB, and RF; only clinical variables for LR and SVM; and clinical + EQ-5D variables for NN.

#### Model Performance

**FIG 5.**
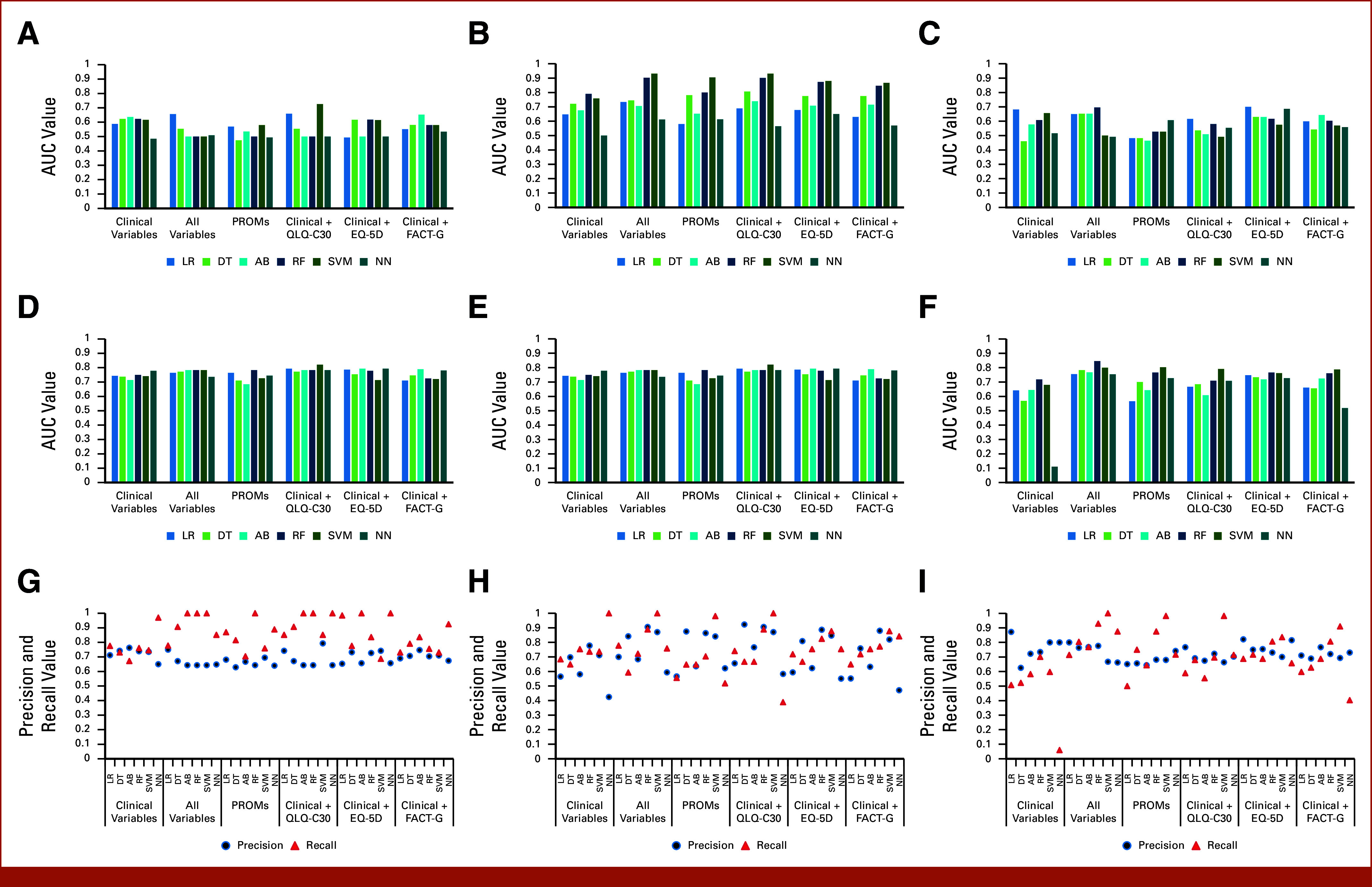
Predicting changes to chemotherapy. AUC values for (A) original, (B) balanced, and (C) partially balanced data sets obtained from all models in all feature sets predicting; F1 scores for (D) original, (E) balanced, and (F) partially balanced data sets obtained from all models in all feature sets predicting; precision and recall distributions for (G) original, (H) balanced, and (I) partially balanced data sets obtained from all models in all feature sets. AB, adaptive boosting; DT, decision tree; EQ-5D, EuroQol Five-Dimensional Visual Analogue Scale; FACT-G, Functional Assessment of Cancer Therapy-General; LR, logistic regression; NN, neural network; PROMs, patient-reported outcome measures; QLQ-C30, European Organisation for Research and Treatment of Cancer Core Quality of Life Questionnaire; RF, random forest; SVMs, support vector machines.

Overall, models predicting changes to chemotherapy performed significantly better than models predicting triage (*P* < .01) and admissions (*P* < .001). No model in original and partially balanced data sets outperformed others. In the balanced data set, the best algorithms were RF and SVM. SVM with all and clinical + QLQ-C30 variables had the best overall performance (AUC = 0.931). The AUCs of the models increased when data were balanced, but there was no difference in F1 scores. There was no noticeable difference between original and partially balanced data sets (Figs 5D and 5F). Model calibration was very good in the partially balanced data set and slightly worse in other data types (Fig 3). LR prioritized clinical variables with individual FACT-G and EQ-5D features. RF mainly considered FACT-G and some clinical variables (Table 1).

## DISCUSSION

We successfully applied a range of ML models to a complex oncology data set with clinical, PROM, and health outcome data. PROMs improved the overall performance of ML models for all target variables. Sometimes the best performing models included only PROM variables. Although there is evidence suggesting that using PROMs without objectively measured data in ML models can lead to accurate predictions,^15^ this study encourages using both clinical and PROM data. The QLQ-C30 questionnaire added the most predictive value overall. This might be explained by QLQ-C30 being the only questionnaire with variables consistently significantly different between classes. These results are promising, as the wide availability of QLQ-C30^40^ may aid its utilization in ML models for clinical practice.

LR being the simplest model and outperforming other methods in imbalanced data was also observed in previous studies.^26,35^ Good performance of RF and SVM when predicting admissions and changes to chemotherapy in balanced data is compatible with ensemble methods of previously reported outcome predictions.^17,18,41^ Changes to chemotherapy predictions had the best overall performance, which is further confirmed by great calibration of models predicting this target in the partially balanced data set. This might be explained by more frequent and stronger significance of feature differences between classes. Poor performance of triage predictions could be due to more subjective nature of this target, compared with clinical decision to admit a patient or make treatment changes. Balancing data sets improved overall model performance. Using the balanced data set might decrease generalizability of models, as oversampling often causes overfitting.^42^ Therefore, evaluating models on the balanced testing set prevents the models from applications in clinical practice, as the real-world data are never perfectly balanced. Nevertheless, training models on imbalanced data can lead to incorrect predictions, biased toward one of the classes,^43^ which was apparent through low recall in admission predictions, being the most imbalanced target. Using the partially balanced data set mitigates such bias and the lack of generalizability. This method ensures robustness of models through the balanced training set and obtains a more accurate perspective for real clinical data through the original testing set.^44^

In all target variables, LR models focused more on clinical features than PROMs. RF models usually favored FACT-G variables with some relevant clinical or QLQ-C30 features (mainly sleep, cognitive, and social scales). Although these patterns were similar for all target variables, changes to chemotherapy predictions showed the smallest discrepancy between the feature ranks. It might be explained by the best predictive performance of this outcome. LR was often the best performing model in original data, which could explain its poor performance of predicting triage and admissions, as the clinical features for these targets did not have significant differences between classes (Appendix Table A3), yet the model was considering these variables the most important (Table 1). For changes to chemotherapy, there were many significantly different clinical features, explaining good performance of LR. RF usually favored PROMs, which explained this model struggling to predict outcomes from only clinical variables.

Inclusion of three different PROMs allowed understanding of their individual predictive value. This study addressed the inconsistency in preprocessing methods for class imbalance in existing studies^18,33,35-37^ and highlighted differences in results generated from these three techniques. The variety of performance metrics reported allowed between-studies comparison^15^ and in-depth understanding of models. Furthermore, consulting clinicians during study design ensured clinical relevance of research questions, which can support adoption of ML methods.^39^

The limitations of this study include clinical trial data collection, which might not be representative of the population.^45^ No information about patients' ethnicity was provided, which limited understanding of potential bias in data^46^ and prevented subgroup analyses.^33^ Small sample size is associated with higher accuracy in classification,^47^ so using more data would prevent potential bias. Furthermore, this work used only PROMs collected at the beginning of chemotherapy (baseline), so potential over-time dependencies of patient reports were missed. Half of the participants used clinical trial intervention, which might have affected the outcome, but this risk was mitigated through performance comparison in control and intervention groups, identifying no significant differences.

In conclusion, this study supported the evidence that PROMs, such as health-related quality of life, functionating, and symptom reporting, can improve the performance of ML models predicting patient outcomes. The predictive value of widely available PROMs, such as the QLQ-C30 questionnaire, supports the motivation for collecting and using these measures in ML research. The results inform further exploration of PROMs' effect as predictors, and potential application of ML models in clinical practice, if rigorous justification and reporting of methodology is performed. On the basis of large discrepancies across results from different preprocessing methods, this research alerts scientific community to justify choices on the methods for balancing data. It is recommended to balance the training set only and to test models on original data to prevent bias. In future work, we plan to involve patients and clinicians to assess their attitudes to ML-based prediction and to explore the broader implications of the findings. We also plan to use PROM data collected longitudinally throughout chemotherapy treatment, as over-time changes in reporting might provide more meaningful conclusions.

## APPENDIX

**TABLE A1.** Variables in Each Feature Sets

Clinical Variable	All Variables	PROMs	Clinical + QLQ-C30	Clinical + EQ-5D	Clinical + FACT-G
**DiseaseSite** Sex **PreviousChemo** **AgeStudyEntry** **PrimaryorMet** **Comorbidities** **DaysonStudy** **StudyArm**	**DiseaseSite** Sex **PreviousChemo** **AgeStudyEntry** **PrimaryorMet** **Comorbidities** **DaysonStudy** **StudyArm** **PhysicalWB Baseline** **SocialWB Baseline** EmotionalWB Baseline **EmotionalWBBaseline** **FunctionalWB Baseline** **QLQ-C30 Appetite 0** **QLQ-C30 Dyspnoea 0** QLQ-C30 Pain 0 QLQ-C30 Fatigue 0 **QLQ-C30 NauseaVom 0** **QLQ-C30 Const 0** **QLQ-C30 Diarr 0** **QLQ-C30 Financ 0** QLQ-C30 GlobalHealth 0 **QLQ-C30 Cognitive 0** **QLQ-C30 Sleep 0** QLQ-C30 Emotional 0 QLQ-C30 Physical 0 QLQ-C30 Role 0 **QLQ-C30 Social 0** **QoLEQ5DMob** **QoLEQ5DSelCar** **QoLEQ5DUsuAct** **QoLEQ5DPain** **QoLEQ5DAnxDep**	**PhysicalWB Baseline** **SocialWB Baseline** EmotionalWB Baseline **FunctionalWB Baseline** **QLQ-C30 Appetite 0** **QLQ-C30 Dyspnoea 0** QLQ-C30 Pain 0 QLQ-C30 Fatigue 0 **QLQ-C30 NauseaVom 0** **QLQ-C30 Const 0** **QLQ-C30 Diarr 0** **QLQ-C30 Financ0** QLQ-C30 GlobalHealth 0 **QLQ-C30 Cognitive 0** **QLQ-C30 Sleep 0** QLQ-C30 Emotional 0 QLQ-C30 Physical 0 QLQ-C30 Role 0 **QLQ-C30 Social 0** **QoLEQ5DMob** **QoLEQ5DSelCar** **QoLEQ5DUsuAct** **QoLEQ5DPain** **QoLEQ5DAnxDep**	**DiseaseSite** Sex **PreviousChemo** **AgeStudyEntry** **PrimaryorMet** **Comorbidities** **DaysonStudy** **StudyArm** **QLQ-C30 Appetite 0** **QLQ-C30 Dyspnoea 0** **QLQ-C30 Pain0** QLQ-C30 Fatigue 0 **QLQ-C30 NauseaVom 0QLQ-C30 Const 0** **QLQ-C30 Diarr 0** **QLQ-C30 Financ 0** QLQ-C30 GlobalHealth 0 **QLQ-C30 Cognitive 0** **QLQ-C30 Sleep 0** **QLQ-C30 Emotional 0** **QLQ-C30 Physical 0** QLQ-C30 Role 0 **QLQ-C30 Social 0**	**DiseaseSite** Sex **PreviousChemo** **AgeStudyEntry** **PrimaryorMet** **Comorbidities** **DaysonStudy** **StudyArm** **QoLEQ5DMob** **QoLEQ5DSelCar** **QoLEQ5DUsuAct** **QoLEQ5DPain** **QoLEQ5DAnxDep**	**DiseaseSite** Sex **PreviousChemo** **AgeStudyEntry** **PrimaryorMet** **Comorbidities** **DaysonStudy** **StudyArm** **PhysicalWB Baseline** **SocialWB Baseline** **FunctionalWB Baseline**

NOTE. Variables in bold were selected in ML models.

Abbreviations: EQ-5D, EuroQol Five-dimensional Visual Analogue Scale; FACT-G, Functional Assessment of Cancer Therapy-General; PROMs, patient-reported outcome measures; QLQ-C30, European Organisation for Research and Treatment of Cancer Core Quality of Life Questionnaire.

**TABLE A2.** Differences in Features Between Classes in All Target Variables and the *P* Value Generated by Using Mann-Whitney *U* Test for All Features but AgeStudyEntry (*t*-test used), as It Was the Only Normally Distributed Variable

Input Variable	Not Admitted (n = 338)		Admitted (n = 170)		Significance Test	
	Median	Mean/Value	Median	Mean/Value	*P*	Significance Level
StudyArm	1	1 = 170, 2 = 168	1	1 = 86, 2 = 84	.475	
DiseaseSite	2	1 = 157, 2 = 58, 3 = 123	2	1 = 76, 2 = 48, 3 = 46	.220	
Sex	2	F = 263, M = 75	2	F = 143, M = 27	.047	*
PreviousChemo	0	No = 266, Yes = 72	0	No = 131, Yes = 39	.337	
AgeStudyEntry	56	55.429	57.5	56.994	.158	
PrimaryorMet	1	1 = 228, 2 = 110	1	1 = 89, 2 = 81	<.001	**
Comorbidities	0	0 = 192, 1 = 103, 2 = 36, 3 = 7	1	0 = 75, 1 = 54, 2 = 30, 3 = 11	<.001	**
DaysonStudy	126	119.891	126	119.888	.118	
PhysicalWB_Baseline	25	23.725	24	22.424	.003	***
SocialWB_Baseline	24	23.693	24	23.552	.126	
EmotionalWB_Baseline	18	16.769	17	16.429	.131	
FunctionalWB_Baseline	19.917	19.016	19	18.263	.123	
C30_Appetite_0	0	16.606	0	27.381	.115	
C30_Dyspnoea_0	0	8.424	0	20.000	.003	***
C30_Pain_0	16.667	20.818	16.667	25.833	.124	
C30_Fatigue_0	22.222	26.835	33.333	36.984	.002	***
C30_NauseaVom_0	0	3.879	0	9.643	.028	*
C30_Const_0	0	12.774	0	15.952	.160	
C30_Diarr_0	0	9.179	0	10.000	.443	
C30_Financ_0	0	15.644	0	13.810	.246	
C30_GlobalHealth_0	75	73.853	75	67.679	.037	*
C30_Cognitive_0	83.333	83.514	83.333	80.595	.250	
C30_Sleep_0	33.333	37.681	33.333	37.619	.388	
C30_Emotional_0	75	73.345	83.333	75.060	.261	
C30_Physical_0	93.333	84.073	80	76.976	.012	*
C30_Role_0	83.333	73.980	83.333	70.513	.103	
C30_Social_0	83.333	74.527	83.333	72.189	.224	
QoLEQ5DMob	1	1.369	1	1.547	.032	*
QoLEQ5DSelCar	1	1.141	1	1.241	.056	
QoLEQ5DUsuAct	1	1.731	2	1.847	.107	
QoLEQ5DPain	2	1.810	2	1.947	.101	
QoLEQ5DAnxDep	2	1.807	2	1.894	.183	

Input Variable	Not Triaged (n = 214)		Triaged (n = 294)		Significance Test	
	Median	Mean/Value	Median	Mean/Value	*P*	Significance Level
StudyArm	2	1 = 104, 2 = 110	1	1 = 152, 2 = 142	.245	
DiseaseSite	2	1 = 85, 2 = 49, 3 = 80	1	1 = 148, 2 = 57, 3 = 89	.011	*
Sex	2	F = 162, M = 52	2	F = 244, M = 50	.022	*
PreviousChemo	0	No = 155, Yes = 59	0	No = 242, Yes = 52	.004	***
AgeStudyEntry	57	56.972	55	55.21	.097	
PrimaryorMet	1	1 = 132, 2 = 82	1	1 = 185, 2 = 109	.388	
Comorbidities	0	0 = 119, 1 = 62, 2 = 25, 3 = 8	0	0 = 148, 1 = 95, 2 = 41, 3 = 10	.136	
DaysonStudy	126	119.575	126	120.119	.489	
PhysicalWB_Baseline	25	23.711	24	22.980	.071	
SocialWB_Baseline	24	23.451	24	23.788	.305	
EmotionalWB_Baseline	18	16.872	18	16.497	.173	
FunctionalWB_Baseline	20	19.088	19	18.527	.154	
C30_Appetite_0	0	17.326	0	22.361	.043	*
C30_Dyspnoea_0	0	10.358	0	13.750	.090	
C30_Pain_0	16.667	19.868	16.667	24.444	.057	
C30_Fatigue_0	22.222	26.962	33.333	32.662	.012	*
C30_NauseaVom_0	0	4.571	0	6.736	.012	*
C30_Const_0	0	15.048	0	12.971	.334	
C30_Diarr_0	0	7.910	0	10.600	.043	*
C30_Financ_0	0	15.443	0	14.722	.358	
C30_GlobalHealth_0	83.333	74.479	75	69.792	.122	
C30_Cognitive_0	83.333	85.217	83.333	80.556	.159	
C30_Sleep_0	33.333	35.028	33.333	39.609	.033	*
C30_Emotional_0	83.333	76.177	75	72.257	.227	
C30_Physical_0	86.667	82.420	86.667	81.153	.493	
C30_Role_0	83.333	75.708	83.333	70.719	.046	*
C30_Social_0	83.333	75.236	83.333	72.660	.234	
QoLEQ5DMob	1	1.458	1	1.409	.344	
QoLEQ5DSelCar	1	1.202	1	1.155	.313	
QoLEQ5DUsuAct	1	1.746	1	1.787	.239	
QoLEQ5DPain	2	1.829	2	1.876	.309	
QoLEQ5DAnxDep	2	1.762	2	1.890	.060	

Input Variable	No Chemotherapy Change (n = 175)		Chemotherapy Change (n = 333)		Significance Test	
	Median	Mean/Value	Median	Mean/Value	*P*	Significance Level
StudyArm	2	1 = 82, 2 = 93	1	1 = 174, 2 = 159	.124	
DiseaseSite	1	1 = 122, 2 = 21, 3 = 32	2	1 = 111, 2 = 85, 3 = 137	<.001	
Sex	2	F = 150, M = 25	2	F = 256, M = 77	.009	***
PreviousChemo	0	No = 145, Yes = 30	0	No = 252, Yes = 81	.032	*
AgeStudyEntry	54	54.126	58	56.913	.011	*
PrimaryorMet	1	1 = 128, 2 = 47	1	1 = 189, 2 = 144	<.001	**
Comorbidities	0	0 = 108, 1 = 52, 2 = 14, 3 = 1	1	0 = 159, 1 = 105, 2 = 52, 3 = 17	<.001	**
DaysonStudy	126	120.211	126	119.721	.085	
PhysicalWB_Baseline	25	23.822	24.5	23.015	.039	*
SocialWB_Baseline	25	24.325	24	23.295	<.001	**
EmotionalWB_Baseline	18	16.662	18	16.653	.346	
FunctionalWB_Baseline	20.5	19.572	19	18.346	.010	*
C30_Appetite_0	0	13.043	0	23.775	.041	*
C30_Dyspnoea_0	0	9.179	0	13.859	.291	
C30_Pain_0	16.667	21.981	16.667	22.760	.377	
C30_Fatigue_0	22.222	25.644	33.333	32.517	.089	
C30_NauseaVom_0	0	3.406	0	7.014	.205	
C30_Const_0	0	10.706	0	15.403	.337	
C30_Diarr_0	0	8.213	0	10.072	.379	
C30_Financ_0	0	16.667	0	14.217	.155	
C30_GlobalHealth_0	83.333	75.302	75	70.024	.014	*
C30_Cognitive_0	83.333	83.454	83.333	82.079	.221	
C30_Sleep_0	33.333	36.983	33.333	37.993	.294	
C30_Emotional_0	75	74.155	75	73.805	.274	
C30_Physical_0	93.333	86.993	86.667	79.068	<.001	**
C30_Role_0	83.333	76.686	66.667	70.796	.032	*
C30_Social_0	83.333	77.168	83.333	71.954	.029	*
QoLEQ5DMob	1	1.276	1	1.511	<.001	**
QoLEQ5DSelCar	1	1.103	1	1.212	.045	*
QoLEQ5DUsuAct	1	1.603	2	1.858	.002	***
QoLEQ5DPain	2	1.776	2	1.899	.046	*
QoLEQ5DAnxDep	2	1.805	2	1.853	.194	

NOTE. Mean, counts of values for categorical variables, and mean for continuous variables are reported.

**P* ≤ .05.

***P* ≤ .001.

****P* ≤ .01.

**TABLE A3.** Results of ANOVA Which Provided *P* < .05 for Outcomes, Preprocessing, and Model Comparisons

Factors Affecting Performance	Methods With Significantly Different AUCs
Outcome	Changes to chemotherapy (0.617)—admissions (0.579)*
	Changes to chemotherapy (0.617)—triage (0.543)**
	Admissions (0.579)—triage (0.543)**
Preprocessing	Balanced (0.671)—original (0.536)**
	Balanced (0.671)—partially balanced (0.533)**
Model	DT (0.604)—NN (0.541)***
	SVM (0.615)—NN (0.541)*

Abbreviations: ANOVA, analysis of variance; DT, decision tree; NN, neural network; SVMs, support vector machines.

**P* ≤ .01.

***P* ≤ .001.

****P* ≤ .05.

**TABLE A4.** Results of the Six Models for Each Target Variable, Each Feature Set, and Each Preprocessing Method Addressing Class Imbalance With Hyperparameters Selected Through Grid Search

Model/Outcome	Input Variable	Original Data						Balanced Data						Balanced Train, Real Test					
		Accuracy	Precision	Recall	F1	AUC	Hyperparameters	Accuracy	Precision	Recall	F1	AUC	Hyperparameters	Accuracy	Precision	Recall	F1	AUC	Hyperparameters
LR																			
Admissions	Clinical variables	0.647	0.667	0.054	0.1	0.513	c = 10, solver = lbfgs	0.654	0.7	0.653	0.676	0.655	c = 10, solver = liblinear	0.569	0.368	0.412	0.389	0.529	c = 1, solver = liblinear
	All variables	0.738	0.476	**0.476**	**0.476**	0.605	c = 100, solver = lbfgs	**0.694**	0.721	**0.721**	**0.721**	**0.691**	c = 100, solver = newton-cg	0.595	0.406	0.464	0.433	0.563	c = 100, solver = lbfgs
	PROMs	**0.75**	0.5	0.238	0.323	0.579	c = 0.01, solver = newton-cg	0.613	0.667	0.59	0.626	0.616	c = 0.1, solver = lbfgs	0.595	0.412	0.5	0.452	0.571	c = 0.01, solver = lbfgs
	Clinical + C30	**0.75**	0.5	**0.476**	0.488	**0.659**	c = 10, solver = liblinear	0.676	**0.727**	0.656	0.69	0.678	c = 100, solver = lbfgs	**0.607**	**0.439**	**0.643**	**0.522**	**0.616**	c = 1, solver = liblinear
	Clinical + EQ-5D	0.637	0	0	0	0.5	c = 0.01, solver = newton-cg	0.64	0.703	0.6	0.647	0.644	c = 100, solver = lbfgs	0.539	0.333	0.382	0.356	0.5	c = 1, solver = liblinear
	Clinical + FACT-G	0.647	**0.545**	0.162	0.25	0.543	c = 1, solver = liblinear	0.632	0.687	0.613	0.648	0.635	c = 1, solver = liblinear	0.578	0.4	0.529	0.456	0.566	c = 100, solver = newton-cg
Triage	Clinical variables	0.588	0.641	0.781	0.704	0.522	c = 100, solver = newton-cg	0.568	0.571	0.6	0.585	0.567	c = 100, solver = lbfgs	0.51	0.567	0.644	0.603	0.485	c = 1, solver = liblinear
	All variables	0.631	0.656	**0.824**	0.73	0.578	c = 10, solver = newton-cg	0.573	0.491	0.65	0.559	0.584	c = 1, solver = liblinear	0.429	0.5	0.375	0.429	0.438	c = 0.1, solver = newton-cg
	PROMs	**0.643**	**0.667**	**0.824**	**0.737**	**0.594**	c = 10, solver = liblinear	**0.594**	0.514	0.475	0.494	0.577	c = 0.1, solver = liblinear	0.571	0.625	0.625	0.625	0.563	c = 0.01, solver = liblinear
	Clinical + C30	0.631	**0.667**	0.784	0.721	0.589	c = 10, solver = liblinear	**0.594**	0.51	0.625	0.562	**0.598**	c = 0.1, solver = liblinear	0.548	0.614	0.563	0.587	0.545	c = 10, solver = liblinear
	Clinical + EQ-5D	0.578	0.644	0.734	0.686	0.525	c = 10, solver = lbfgs	0.508	0.515	0.583	0.547	0.507	c = 1, solver = liblinear	0.549	0.6	**0.661**	0.629	0.528	c = 10, solver = newton-cg
	Clinical + FACT-G	0.598	0.646	0.797	0.713	0.53	c = 10, solver = lbfgs	0.585	**0.577**	**0.683**	**0.626**	0.583	c = 0.1, solver = newton-cg	**0.608**	**0.694**	0.576	**0.63**	**0.624**	c = 100, solver = lbfgs
Chemo	Clinical variables	0.647	0.712	0.776	0.743	0.588	c = 1, solver = liblinear	0.642	0.565	0.684	0.619	0.647	c = 0.1, solver = liblinear	0.627	**0.872**	0.507	0.642	**0.682**	c = 0.1, solver = newton-cg
	All variables	0.69	**0.75**	0.778	0.764	0.656	c = 10, solver = newton-cg	**0.732**	**0.7**	**0.778**	**0.737**	**0.734**	c = 1, solver = liblinear	0.69	0.8	**0.714**	**0.755**	0.649	c = 10, solver = liblinear
	PROMs	0.655	0.681	0.87	0.764	0.569	c = 1, solver = liblinear	0.58	0.566	0.556	0.561	0.58	c = 0.01, solver = newton-cg	0.488	0.651	0.5	0.566	0.482	c = 0.1, solver = lbfgs
	Clinical + C30	**0.714**	0.742	0.852	**0.793**	**0.659**	c = 10, solver = liblinear	0.688	0.656	0.741	0.696	0.689	c = 1, solver = liblinear	0.607	0.767	0.589	0.667	0.616	c = 1, solver = lbfgs
	Clinical + EQ-5D	0.647	0.653	**0.985**	0.786	0.493	c = 0.01, solver = lbfgs	0.672	0.594	0.719	0.651	0.678	c = 0.1, solver = lbfgs	**0.696**	0.821	0.687	0.748	0.7	c = 0.1, solver = newton-cg
	Clinical + FACT-G	0.608	0.69	0.731	0.71	0.551	c = 1, solver = newton-cg	0.627	0.552	0.649	0.597	0.63	c = 0.1, solver = liblinear	0.598	0.71	0.597	0.661	0.599	c = 0.1, solver = newton-cg
DT																			
Admissions	Clinical variables	0.618	0.25	0.027	0.049	0.49	gini, 2,2,2	0.75	0.773	0.773	0.773	0.747	log_loss, 14,2,4	0.569	0.308	0.235	0.267	0.485	gini, 14,2,2
	All variables	**0.738**	0.444	0.19	0.267	0.556	entropy, 2,14,2	**0.793**	**0.779**	0.869	0.822	0.784	log_loss, 12,2,4	0.583	0.36	0.321	0.34	0.518	entropy, 12,2,8
	PROMs	**0.738**	0.444	0.19	0.267	0.556	gini, 2,4,2	0.82	0.847	0.82	**0.833**	0.82	gini, 18,2,2	0.607	0.4	0.357	0.377	0.545	log_loss, 18,2,4
	Clinical + C30	0.631	0.321	**0.429**	0.367	**0.563**	entropy, 8,18,14	0.82	0.86	0.803	0.831	**0.822**	gini, 20,2,8	**0.619**	**0.433**	**0.464**	**0.448**	**0.58**	gini, 12,4,2
	Clinical + EQ-5D	0.618	**0.464**	0.351	**0.4**	0.56	gini, 20,2,20	0.757	0.733	**0.88**	0.8	0.743	entropy, 14,2,2	0.588	0.395	0.441	0.417	0.551	entropy, 20,4,2
	Clinical + FACT-G	0.578	0.412	0.378	0.394	0.535	entropy, 18,4,2	0.779	0.785	0.827	0.805	0.774	gini, 14,2,2	0.578	0.32	0.235	0.271	0.493	gini, 18,2,2
Triage	Clinical variables	0.549	0.615	0.75	0.676	0.48	gini, 2,2,2	0.61	0.652	0.5	0.566	0.612	log_loss, 18,2,4	0.51	0.576	0.576	0.576	0.497	entropy, 20,2,4
	All variables	0.583	0.643	0.706	0.673	0.55	gini, 12,18,14	**0.698**	0.641	0.625	0.633	**0.688**	gini, 18,2,4	0.5	0.554	0.646	0.596	0.476	entropy, 20,2,2
	PROMs	**0.607**	**0.688**	0.647	0.667	**0.596**	log_loss, 18,4,2	0.604	0.525	0.525	0.525	0.593	gini, 12,2,2	0.571	**0.65**	0.542	0.591	0.576	gini, 12,2,2
	Clinical + C30	0.488	0.611	0.431	0.506	0.504	log_loss, 12,12,5	0.677	0.615	0.6	0.608	0.666	log_loss, 14,2,4	0.524	0.574	0.646	0.608	0.503	log_loss, 18,2,4
	Clinical + EQ-5D	0.52	0.619	0.609	0.614	0.489	gini, 12,2,18	0.661	0.717	0.55	0.623	0.663	entropy, 20,2,2	0.539	0.603	0.593	0.598	0.529	log_loss, 20,2,2
	Clinical + FACT-G	0.598	0.635	**0.844**	**0.725**	0.514	entropy, 4,2,4	0.669	**0.691**	**0.633**	**0.661**	0.67	entropy, 20,2,2	**0.598**	0.645	**0.678**	**0.661**	**0.583**	gini, 18,2,4
Chemo	Clinical variables	0.657	**0.742**	0.731	0.737	**0.623**	log_loss, 4,14,4	0.731	0.698	0.649	0.673	0.721	log_loss, 12,4,14	0.48	0.625	0.522	0.569	0.461	gini, 20,2,12
	All variables	0.655	0.671	**0.907**	**0.772**	0.554	gini, 2,8,2	0.75	0.842	0.593	0.696	0.745	log_loss, 18,2,2	**0.702**	**0.763**	**0.804**	**0.783**	**0.652**	gini, 20,2,2
	PROMs	0.571	0.629	0.815	0.71	0.474	entropy, 12,4,20	0.786	0.875	0.648	0.745	0.781	entropy, 20,2,2	0.571	0.656	0.75	0.7	0.482	gini, 14,2,4
	Clinical + C30	0.655	0.671	**0.907**	**0.772**	0.554	entropy, 2,8,2	**0.813**	**0.923**	0.667	**0.774**	**0.807**	log_loss, 14,4,2	0.583	0.691	0.679	0.685	0.536	entropy, 20,2,4
	Clinical + EQ-5D	**0.667**	0.732	0.776	0.754	0.617	entropy, 4,18,2	0.791	0.809	0.667	0.731	0.775	gini, 12,2,2	0.657	0.75	0.716	0.733	0.63	entropy, 20,2,2
	Clinical + FACT-G	0.647	0.707	0.791	0.746	0.581	log_loss, 4,18,2	0.784	0.759	**0.719**	0.739	0.775	entropy, 14,4,2	0.569	0.689	0.627	0.656	0.542	log_loss, 18,2,4
AB																			
Admissions	Clinical variables	0.618	0.25	0.027	0.049	0.49	SAMME, 1.0, est = 10	0.566	0.629	0.52	0.569	0.571	SAMME.R, 1.0, est = 500	0.5	0.257	0.265	0.261	0.441	SAMME.R, 1.0, est = 500
	All variables	**0.762**	**0.667**	0.095	0.167	0.54	SAMME, 0.01, est = 500	0.676	0.687	**0.754**	0.719	0.667	SAMME.R, 1.0, est = 100	**0.643**	**0.469**	**0.536**	**0.5**	**0.616**	SAMME.R, 1.0, est = 500
	PROMs	0.738	0.333	0.048	0.083	0.508	SAMME, 0.01, est = 500	0.649	0.683	0.672	0.678	0.646	SAMME.R, 1.0, est = 500	0.548	0.353	0.429	0.387	0.518	SAMME.R, 1.0, est = 500
	Clinical + C30	0.655	0.3	**0.286**	**0.293**	**0.532**	SAMME.R, 1.0, est = 50	**0.739**	**0.767**	**0.754**	**0.76**	**0.737**	SAMME.R, 0.1, est = 500	0.595	0.417	0.536	0.469	0.58	SAMME.R, 1, est = 500
	Clinical + EQ-5D	0.618	0.25	0.027	0.049	0.49	SAMME, 1.0, est = 50	0.61	0.683	0.547	0.607	0.618	SAMME.R, 1.0, est = 500	0.461	0.216	0.235	0.225	0.404	SAMME.R, 1.0, est = 500
	Clinical + FACT-G	0.627	0	0	0	0.492	SAMME.R, 0.01, est = 100	0.684	0.716	0.707	0.711	0.681	SAMME.R, 1.0, est = 500	0.48	0.229	0.235	0.232	0.419	SAMME.R, 1.0, est = 500
Triage	Clinical variables	0.569	0.622	0.797	0.699	0.491	SAMME.R, 0.01, est = 500	0.568	**0.585**	0.517	0.549	0.569	SAMME.R, 1.0, est = 500	0.51	0.579	0.559	0.569	0.501	SAMME.R, 0.1, est = 100
	All variables	0.512	0.586	0.667	0.624	0.47	SAMME.R, 0.1, est = 500	**0.667**	0.583	**0.7**	**0.636**	**0.671**	SAMME.R, 1.0, est = 500	0.476	0.543	0.521	0.532	0.469	SAMME.R, 1.0, est = 500
	PROMs	0.524	0.604	0.627	0.615	0.496	SAMME.R, 1.0, est = 500	0.646	0.56	**0.7**	0.622	0.654	SAMME.R, 1.0, est = 500	0.464	0.532	0.521	0.526	0.455	SAMME.R, 1.0, est = 500
	Clinical + C30	**0.607**	0.61	**0.98**	**0.752**	**0.505**	SAMME.R, 0.1, est = 10	0.646	0.571	0.6	0.585	0.639	SAMME.R, 1.0, est = 10	0.524	0.583	0.583	0.583	0.514	SAMME.R, 1.0, est = 500
	Clinical + EQ-5D	0.578	**0.63**	0.797	0.703	0.504	SAMME, 0.01, est = 100	0.525	0.536	0.5	0.517	0.526	SAMME.R, 1.0, est = 500	**0.578**	**0.618**	**0.712**	**0.661**	**0.554**	SAMME.R, 0.1, est = 500
	Clinical + FACT-G	0.578	**0.63**	0.797	0.703	0.504	SAMME, 1.0, est = 10	0.585	0.59	0.6	0.595	0.584	SAMME.R, 1.0, est = 500	0.49	0.557	0.576	0.567	0.474	SAMME.R, 1.0, est = 500
Chemo	Clinical variables	0.647	**0.763**	0.672	0.714	0.636	SAMME.R, 0.01, est = 500	0.664	0.581	**0.754**	0.646	0.676	SAMME, 1.0, est = 500	0.578	0.722	0.582	0.645	0.577	SAMME.R, 1.0, est = 50
	All variables	0.643	0.643	**1**	0.783	0.5	SAMME.R, 0.1, est = 10	0.705	0.684	0.722	0.703	0.706	SAMME, 1, est = 500	**0.69**	**0.768**	**0.768**	**0.768**	**0.652**	SAMME.R, 1.0, est = 500
	PROMs	0.583	0.667	0.704	0.685	0.535	SAMME.R, 1.0, est = 50	0.652	0.636	0.648	0.642	0.652	SAMME.R, 1.0, est = 100	0.524	0.643	0.643	0.643	0.464	SAMME.R, 0.1, est = 500
	Clinical + C30	0.643	0.643	**1**	0.783	0.5	SAMME, 0.1, est = 100	**0.741**	**0.766**	0.667	**0.713**	**0.739**	SAMME.R, 1.0, est = 500	0.524	0.674	0.554	0.608	0.509	SAMME.R, 1.0, est = 500
	Clinical + EQ-5D	0.657	0.657	**1**	**0.793**	0.5	SAMME, 0.1, est = 50	0.701	0.623	**0.754**	0.683	0.708	SAMME.R, 1.0, est = 100	0.647	0.754	0.687	0.719	0.629	SAMME.R, 1.0, est = 500
	Clinical + FACT-G	**0.706**	0.747	0.836	0.789	**0.646**	SAMME, 1, est = 100	0.709	0.632	**0.754**	0.688	0.715	SAMME.R, 0.1, est = 500	0.657	0.767	0.687	0.724	0.643	SAMME.R, 1.0, est = 100
RF																			
Admissions	Clinical variables	0.637	0	0	0	0.5	entropy, 2,2 est = 100	0.816	0.813	0.867	0.839	0.81	gini, 14,2, est = 100	0.471	0.25	0.294	0.27	0.426	gini, 14,2, est = 100
	All variables	0.702	0.333	0.19	0.242	0.532	gini, 2,8, est = 10	0.901	0.891	0.934	0.912	0.897	gini, 14,2, est = 500	**0.69**	**0.583**	0.25	0.35	0.58	log_loss, 14,2, est = 100
	PROMs	0.726	0.375	0.143	0.207	0.532	log_loss, 18,4, est = 10	0.865	0.838	0.934	0.884	0.857	gini, 18,2, est = 100	0.655	0.478	**0.393**	**0.431**	**0.589**	entropy, 18,2, est = 100
	Clinical + C30	**0.762**	**0.545**	**0.286**	**0.375**	**0.603**	entropy, 14,2 est = 100	**0.91**	**0.892**	**0.951**	**0.921**	**0.905**	gini, 14,2, est = 100	0.655	0.478	**0.393**	**0.431**	**0.589**	gini, 18,2, est = 100
	Clinical + EQ-5D	0.627	0.455	0.135	0.208	0.521	log_loss, 8,18 est = 10	0.794	0.783	0.867	0.823	0.786	entropy, 18,2, est = 500	0.539	0.276	0.235	0.254	0.463	gini, 18,2, est = 500
	Clinical + FACT-G	0.627	0.471	0.216	0.296	0.539	gini, 18,14, est = 100	0.868	0.87	0.893	0.882	0.865	log_loss, 18,2, est = 100	0.667	0.5	0.206	0.292	0.551	gini, 18,2, est = 100
Triage	Clinical variables	0.588	0.625	0.859	0.724	0.495	log_loss, 2,18, est = 500	0.61	0.63	0.567	0.597	0.611	entropy, 18,2, est = 500	0.471	0.536	0.627	0.578	0.441	entropy, 18,2, est = 500
	All variables	0.512	0.596	0.608	0.602	0.486	entropy, 18,18, est = 10	**0.719**	0.633	**0.775**	0.697	**0.727**	gini, 18,2, est = 100	0.5	0.552	0.667	0.604	0.472	gini, 18,2, est = 500
	PROMs	**0.607**	0.615	**0.941**	**0.744**	**0.516**	gini, 2,18, est = 10	0.677	0.592	0.725	0.652	0.684	log_loss, 18,2, est = 500	0.5	0.545	0.75	0.632	0.458	entropy, 14,2, est = 500
	Clinical + C30	0.536	0.604	0.686	0.642	0.495	log_loss, 8,14, est = 10	0.698	0.604	0.8	0.688	0.713	gini, 18,2, est = 500	0.5	0.55	0.667	0.604	0.472	gini, 18,2, est = 100
	Clinical + EQ-5D	0.598	**0.635**	0.844	0.725	0.514	entropy, 2,14, est = 10	0.703	**0.705**	0.717	**0.711**	0.703	gini, 18,2, est = 100	**0.618**	**0.645**	**0.847**	**0.719**	**0.575**	entropy, 14,2, est = 100
	Clinical + FACT-G	0.608	0.633	0.891	0.74	0.511	entropy, 2,18, est = 10	0.686	0.695	0.683	0.689	0.686	log_loss, 18,2, est = 100	0.588	0.605	0.831	0.7	0.543	gini, 14,2, est = 500
Chemo	Clinical variables	0.667	**0.739**	0.761	0.75	**0.623**	log_loss, 8,18, est = 100	0.799	0.778	0.737	0.757	0.79	log_loss, 14,2, est = 500	0.637	0.734	0.701	0.718	0.608	gini, 18,2, est = 500
	All variables	0.643	0.643	**1**	**0.783**	0.5	log_loss, 2,18 est = 10	**0.902**	**0.906**	**0.889**	**0.897**	**0.903**	entropy, 14,2, est = 500	**0.774**	**0.776**	**0.929**	**0.846**	**0.696**	gini, 14,2, est = 500
	PROMs	0.643	0.643	**1**	**0.783**	0.5	log_loss, 2,18 est = 10	0.804	0.864	0.704	0.776	0.8	log_loss, 14,2, est = 100	0.643	0.681	0.875	0.766	0.527	log_loss, 18,2, est = 500
	Clinical + C30	0.643	0.643	**1**	**0.783**	0.5	entropy, 2,14, est = 10	**0.902**	**0.906**	**0.889**	**0.897**	0.901	entropy, 18,2, est = 500	0.619	0.722	0.696	0.709	0.58	entropy, 18,2, est = 500
	Clinical + EQ-5D	**0.686**	0.727	0.836	0.778	0.618	gini, 14,18, est = 100	0.881	0.887	0.825	0.855	0.873	entropy, 18,2, est = 500	0.676	0.73	0.806	0.766	0.617	gini, 18,2, est = 100
	Clinical + FACT-G	0.627	0.704	0.756	0.725	0.573	entropy, 14,14, est = 10	0.858	0.88	0.772	0.822	0.847	log_loss, 14,2, est = 100	0.667	0.72	0.806	0.761	0.603	gini, 18,2, est = 500
SVM																			
Admissions	Clinical variables	0.637	0	0	0	0.5	c = 0.01, gamma = 0.001, kernel = linear	0.75	0.766	0.787	0.776	0.746	c = 1, gamma = 1, kernel = rbf	0.49	0.2	**0.176**	**0.188**	0.411	c = 1, gamma = 1, kernel = rbf
	All variables	0.738	0.462	0.286	0.353	0.587	c = 0.1, gamma = 0.001, kernel = linear	**0.892**	**1**	**0.803**	**0.891**	**0.902**	c = 1, gamma = 0.1, kernel = rbf	**0.667**	0	0	0	0.5	c = 0.1, gamma = 1, kernel = rbf
	PROMs	0.726	0.333	0.095	0.148	0.516	c = 1, gamma = 0.01, kernel = rbf	0.45	0	0	0	0.5	c = 0.1, gamma = 1, kernel = rbf	**0.667**	**0.5**	0.036	0.067	0.509	c = 1, gamma = 1, kernel = rbf
	Clinical + C30	**0.75**	**0.5**	**0.286**	**0.364**	**0.595**	c = 1, gamma = 0.001, kernel = linear	0.874	**1**	0.77	0.87	0.885	c = 1, gamma = 0.01, kernel = rbf	**0.667**	0	0	0	0.5	c = 1, gamma = 1, kernel = rbf
	Clinical + EQ-5D	0.637	0	0	0	0.5	c = 0.01, gamma = 0.01, kernel = rbf	0.787	0.859	0.733	0.791	0.793	c = 1, gamma = 1, kernel = rbf	0.618	0.273	0.088	0.133	0.485	c = 1, gamma = 1, kernel = rbf
	Clinical + FACT-G	0.637	0	0	0	0.5	c = 0.1, gamma = 0.001, kernel = poly	0.838	0.896	0.8	0.845	0.843	c = 1, gamma = 1, kernel = rbf	0.647	0.429	**0.176**	0.25	**0.529**	c = 1, gamma = 1, kernel = rbf
Triage	Clinical variables	0.559	0.62	0.766	0.685	0.488	c = 1, gamma = 0.01, kernel = linear	0.559	0.554	0.683	0.612	0.557	c = 1, gamma = 0.1, kernel = rbf	0.49	0.554	0.61	0.581	0.468	c = 1, gamma = 1, kernel = rbf
	All variables	**0.643**	**0.662**	0.843	0.741	**0.588**	c = 1, gamma = 0.001, kernel = linear	0.729	0.609	0.975	0.75	**0.764**	c = 1, gamma = 1, kernel = rbf	**0.583**	0.578	1	**0.733**	0.514	c = 1, gamma = 1, kernel = rbf
	PROMs	0.619	0.642	0.843	0.729	0.558	c = 1, gamma = 0.001, kernel = linear	0.417	0.417	**1**	0.588	0.5	c = 0.1, gamma = 1, kernel = rbf	**0.583**	0.58	**0.979**	0.729	0.517	c = 0.1, gamma = 1, kernel = rbf
	Clinical + C30	0.607	0.607	**1**	**0.756**	0.5	c = 0.1, gamma = 0.001, kernel = sigmoid	0.688	0.589	0.825	0.686	0.707	c = 1, gamma = 0.1, kernel = rbf	0.56	0.566	**0.979**	0.718	0.49	c = 0.01, gamma = 1, kernel = rbf
	Clinical + EQ-5D	0.549	0.618	0.734	0.671	0.486	c = 0.01, gamma = 0.01, kernel = poly	**0.763**	**0.729**	0.85	0.785	0.761	c = 1, gamma = 1, kernel = rbf	0.578	0.593	0.864	0.703	**0.525**	c = 1, gamma = 1, kernel = rbf
	Clinical + FACT-G	0.569	0.632	0.75	0.686	0.507	c = 0.1, gamma = 0.01, kernel = poly	0.746	0.688	0.917	**0.786**	0.743	c = 1, gamma = 1, kernel = rbf	0.578	0.585	0.932	0.719	0.513	c = 1, gamma = 1, kernel = rbf
Chemo	Clinical variables	0.657	0.735	0.746	0.741	0.616	c = 1, gamma = 0.1, kernel = rbf	0.761	0.712	0.737	0.724	0.758	c = 1, gamma = 1, kernel = rbf	0.637	**0.8**	0.597	0.68	**0.656**	c = 1, gamma = 1, kernel = rbf
	All variables	0.643	0.643	**1**	0.783	0.5	c = 0.01, gamma = 0.001, kernel = linear	**0.929**	**0.871**	**1**	**0.931**	**0.931**	c = 1, gamma = 1, kernel = rbf	0.667	0.667	**1**	0.8	0.5	c = 1, gamma = 1, kernel = rbf
	PROMs	0.631	0.695	0.759	0.726	0.58	c = 1, gamma = 0.001, kernel = poly	0.902	0.841	0.981	0.906	0.905	c = 1, gamma = 1, kernel = rbf	**0.679**	0.679	0.982	**0.803**	0.527	c = 1, gamma = 1, kernel = rbf
	Clinical + C30	**0.762**	**0.793**	0.852	**0.821**	**0.726**	c = 1, gamma = 0.001, kernel = linear	**0.929**	**0.871**	**1**	**0.931**	**0.931**	c = 1, gamma = 1, kernel = rbf	0.655	0.663	0.982	0.791	0.491	c = 1, gamma = 1, kernel = rbf
	Clinical + EQ-5D	0.637	0.742	0.687	0.713	0.615	c = 0.1, gamma = 0.001, kernel = poly	0.881	0.847	0.877	0.862	0.88	c = 1, gamma = 1, kernel = rbf	0.657	0.7	0.836	0.762	0.575	c = 1, gamma = 1, kernel = rbf
	Clinical + FACT-G	0.627	0.71	0.731	0.721	0.58	c = 0.1, gamma = 0.01, kernel = poly	0.866	0.82	0.877	0.847	0.867	c = 1, gamma = 1, kernel = rbf	0.676	0.693	0.91	0.787	0.57	c = 1, gamma = 1, kernel = rbf
NN																			
Admissions	Clinical variables	0.637	0	0	0	0.5	relu, 0.00001, (150,60,30), adam	0.493	0.615	0.213	0.317	0.525	tanh, 0.00001, (100,50,20), adam	0.333	0.33	0.97	**0.493**	0.493	tanh, 0.00001, (150,60,30), adam
	All variables	**0.726**	**0.4**	0.19	0.258	0.548	tanh, 0.001, (100,50,20), adam	0.73	0.763	0.738	0.75	0.729	tanh, 0.0001, (150,60,30), adam	**0.643**	**0.438**	0.25	0.318	0.545	tanh, 0.001, (150,60,30), adam
	PROMs	0.667	0.333	**0.333**	**0.333**	**0.556**	relu, 0.01, (100), adam	0.685	0.717	0.705	0.711	0.682	tanh, 0.0001, (150,60,30), adam	0.548	0.273	0.214	0.24	0.464	tanh, 0.001, (150,60,30), adam
	Clinical + C30	0.702	0.25	0.095	0.138	0.5	tanh, 0.01, (100,50,20), adam	**0.766**	**0.787**	0.787	**0.787**	**0.763**	tanh, 0.00001, (150,60,30), adam	0.607	0.407	0.393	0.4	**0.554**	tanh, 0.0001, (100,50,20), adam
	Clinical + EQ-5D	0.637	0	0	0	0.5	relu, 0.001, (100), sgd	0.625	0.607	**0.907**	0.727	0.593	tanh, 0.001, (150,60,30), adam	0.52	0.347	0.5	0.41	0.515	tanh, 0.001, (150,60,30), adam
	Clinical + FACT-G	0.647	1	0.027	0.053	0.514	tanh, 0.0001, (100,50,20), sgd	0.537	0.7	0.28	0.4	0.566	tanh, 0.01, (150,60,30), adam	0.333	0.333	**1**	0.5	0.5	tanh, 0.01, (100,50,20), adam
Triage	Clinical variables	0.627	0.627	**1**	**0.771**	0.5	tanh, 0.001, (100,50,20), sgd	0.492	0	0	0	0.5	tanh, 0.001, (100,50,20), adam	0.569	0.579	0.932	0.714	0.501	tanh, 0.0001, (150,60,30), adam
	All variables	**0.631**	**0.628**	0.961	0.76	**0.541**	relu, 0.0001, (100), adam	**0.573**	0.486	0.425	0.453	0.552	tanh, 0.0001, (150,60,30), adam	0.464	0.543	0.396	0.458	0.476	tanh, 0.001, (150,60,30), adam
	PROMs	0.548	0.61	0.706	0.655	0.504	relu, 0.01, (150,60,30), sgd	0.563	0.471	0.4	0.432	0.539	relu, 0.0001, (150,60,30), adam	0.536	0.6	0.563	0.581	0.531	tanh, 0.00001, (150,60,30), adam
	Clinical + C30	0.56	0.613	0.745	0.673	0.509	relu, 0.01, (150,60,30), sgd	0.427	0.405	0.8	0.538	0.48	tanh, 0.01, (150,60,30), adam	0.524	0.574	0.646	0.608	0.503	tanh, 0.001, (150,60,30), adam
	Clinical + EQ-5D	0.618	0.624	0.984	0.764	0.492	relu, 0.0001, (150,60,30), adam	0.559	**0.6**	0.4	0.48	**0.56**	tanh, 0.01, (150,60,30), adam	**0.598**	**0.598**	0.932	0.728	**0.536**	tanh, 0.001, (150,60,30), adam
	Clinical + FACT-G	0.627	0.627	**1**	**0.771**	0.5	relu, 0.001, (100), sgd	0.483	0.495	**0.883**	**0.635**	0.476	tanh, 0.0001, (100), adam	0.588	0.584	**1**	**0.737**	0.512	tanh, 0.0001, (100,50,20), sgd
Chemo	Clinical variables	0.637	0.65	0.97	0.778	0.485	relu, 0.01, (100,50,20), sgd	0.425	0.425	**1**	0.597	0.5	tanh, 0.01, (150,60,30), adam	0.373	0.8	0.06	0.111	0.516	tanh, 0.001, (100), agd
	All variables	0.607	0.648	0.852	0.736	0.509	relu, 0.0001, (150,60,30), sgd	0.607	0.594	0.759	**0.651**	0.612	tanh, 0.001, (150,60,30), adam	0.619	0.662	**0.875**	**0.754**	0.491	tanh, 0.001, (150,60,30), adam
	PROMs	0.607	0.64	0.889	0.744	0.494	tanh, 0.001, (100), adam	0.616	**0.622**	0.519	0.566	0.613	tanh, 0.001, (150,60,30), adam	0.643	0.741	0.716	0.727	0.607	tanh, 0.001, (150,60,30), adam
	Clinical + C30	0.643	0.643	**1**	0.783	0.5	relu, 0.001, (100), sgd	0.571	0.583	0.389	0.467	0.565	tanh, 0.01, (150,60,30), adam	0.607	0.702	0.714	0.708	0.554	tanh, 0.001, (150,60,30), adam
	Clinical + EQ-5D	**0.657**	0.657	**1**	**0.793**	0.5	relu, 0.00001, (100,50,20), sgd	**0.634**	0.551	0.754	0.637	**0.65**	tanh, 0.0001, (150,60,30), adam	**0.676**	**0.815**	0.657	0.727	**0.686**	tanh, 0.0001, (150,60,30), adam
	Clinical + FACT-G	**0.657**	**0.674**	0.925	0.78	**0.534**	tanh, 0.001, (100,50,20), adam	0.53	0.471	0.842	0.604	0.57	tanh, 0.00001, (150,60,30), adam	0.51	0.73	0.403	0.519	0.559	tanh, 0.001, (150,60,30), adam

NOTE. Hyperparameters considered for LR were regularization strength and solver. Hyperparameters considered for DT were criterion, maximum depth, the minimum number samples required to split, and minimum number of samples required to be at a leaf node. Hyperparameters considered for AB were number of estimators, learning rate, and boosting algorithm. Hyperparameters considered for RF were criterion, maximum depth, the minimum number samples required to split, and the number of estimators. Hyperparameters considered for SVM were kernel type, kernel coefficient, and regularization parameter. Hyperparameters considered for NN were activation function for the hidden layer, solver for weight optimization, the number of neurons in hidden layers, and the strength of the L2 regularization term.

Abbreviations: AB, adaptive boosting; DT, decision tree; EQ-5D, EuroQol Five-Dimensional Visual Analogue Scale; FACT-G, Functional Assessment of Cancer Therapy-General; LR, logistic regression; NN, neural network; PROMs, patient-reported outcome measures; QLQ-C30, European Organisation for Research and Treatment of Cancer Core Quality of Life Questionnaire; RF, random forest; SVM, support vector machine. Highest values achieved by all models in all target variables are set in bold.
